# A practical approach to adult acute respiratory distress syndrome

**DOI:** 10.4103/0972-5229.76084

**Published:** 2010

**Authors:** Constantine A. Manthous

**Affiliations:** **From:** Yale University School of Medicine, Bridgeport Hospital and Yale University School of Medicine, 267 Grant Street, Bridgeport, CT 06610, USA

**Keywords:** Acute respiratory distress syndrome, mechanical ventilation, positive end expiratory pressure

## Abstract

Acute respiratory distress syndrome (ARDS) is a common disease encountered in hospitalized adult patients that, historically, has carried a very high mortality. This article reviews the clinical features and how pathophysiology informs the evidence-based management of ARDS.

In the landmark *Crossing the Quality Chasm*, authors from the Institute of Medicine argue that physicians take far too long to apply evidence-based techniques to routine patient care.[1] Despite a landmark study describing the only intervention ever shown to reduce mortality (by 22%) in ARDS, implementation was poor as recently as 2004.[2,3] This article reviews briefly the pathogenesis and definitions of ARDS, and reviews evidence-based strategies to improve patients’ outcomes.

## Definition and Pathogenesis

Acute respiratory distress syndrome (ARDS) is a relatively common illness, occurring in 22.4 cases per 105 patient-years, i.e. 150,000 cases each year in the USA[4,5] ARDS is defined as “as syndrome of inflammation and increased permeability” leading to acute onset of bilateral infiltrates on chest radiograph with PaO_2_/FiO_2_≤200 that cannot be explained by left heart dysfunction (i.e. pulmonary artery occlusion pressure>18 mmHg).[5] To the extent that bilateral pneumonia, some interstitial lung diseases and even lung hemorrhage can meet these criteria, ARDS is a descriptive term that includes a variety of pathogenic processes. Most animal models of ARDS employ insults to cause diffuse alveolar damage (DAD), so many clinicians and investigators think of ARDS as DAD. Readers should be aware that “not all ARDS is the same” because of the poor specificity associated with the working definition. The author uses the term ARDS to designate a diffuse inflammatory process that spares little/no lung and almost always requires positive end-expiratory pressure >5 cmH_2_O to reverse hypoxemia. The reason is that most of the accumulated science on management of ARDS applies to this lesion much more than to bilateral focal lung processes. Acute lung injury (ALI), which includes ARDS, includes all such patients with PaO_2_/FiO_2_≤300 and implies that lung injury/ARDS includes a spectrum of severity.

ARDS is an inflammatory lung lesion triggered by a multitude of insults that activate host immunologic mechanisms. Sepsis, especially pneumonia, is by far the most common cause.[6] But other injuries, including major trauma, burns, electrical injuries, drowning, pancreatitis, inhaled or injected toxins, and transfusions (TRALI) may cause ALI.[7] It is also worth noting here that the precise immunologic mechanisms that trigger ALI vary from cause-to-cause. However, some features are implicit in models of DAD. In the classic model, an inflammatory trigger causes recruitment of neutrophils to the capillary membrane through elaboration of surface adhesion molecules. Once “snagged,” neutrophils degranulate causing direct injury of the capillary membrane. If of sufficient severity, these inflammatory events increase microvascular permeability to salt, water, and macromolecules that egress into the interstitium, eventually overwhelming clearance mechanisms to flood alveoli. Proteins in the leaked fluid may coagulate appearing as the classic hyaline membranes (on pathologic specimens) of early DAD. Meanwhile a variety of inflammatory mediators may incite apoptosis of type II pneumocytes that produce surfactant. Surfactant depletion promotes atelectasis that is most prominent in dependent lung regions, i.e. posteriorly in a supine patient.[8,9] So ARDS is rightly conceived as a syndrome of both flooding and atelectasis---a seminal concept for management. In the first 7 days, capillary leak, atelectasis, and acute cellular inflammation predominate, termed the “exudative phase” which is typified by shunt physiology. If the inciting mechanism is addressed and additional pro-inflammatory events prevented, many patients will heal with minimal residua. However, starting on roughly days 7-10, acute cellular inflammation wanes and is replaced by cells of chronic inflammation including fibroblasts that produce collagen, termed the “fibroproliferative phase.” While it is not certain, the duration and severity of the initial insult and/or genetic factors of the host may determine who goes on to develop this fibroproliferative phase, marked by waning shunt and increasing dead space as the lung fibroses.[10]

Before recent advances, mortality of patients with ARDS exceeded 40%, but with greater understanding of the pathophysiology and translation to bedside management, (if we can “bridge the quality chasm” by implementing life-saving techniques) mortality should decrease in the 21st century.

## Management

There are two fundamental tenets to maximally manage patients with ARDS:

1. Identify and reverse the inflammatory cause as quickly as possible. The duration and magnitude of the initial insult determines, with perhaps genomic predisposition, the severity and duration of ARDS. Especially with sepsis, clinicians should seek to eradicate infections rapidly, including interventional/surgical drainage of loculated foci that will not otherwise respond to antibiotics alone.[10]
2. Avoid introduction of pro-inflammatory iatrogenesis that will maintain the lesion. Historically, the greatest insult that promoted ongoing ARDS was mechanical ventilation with normal or high tidal volumes that “baro-traumatized” the already injured lung (termed ventilator-induced lung injury; VILI). However a variety of relatively common iatrogenic stimuli, including transfusions, nosocomial infections (especially of lines and lung, but can be of any site) and drugs can promote additional injury and non-healing. This model---called the “multiple hit hypothesis”---posits that while ARDS may be initiated by one insult, it is often maintained by readily preventable pro-inflammatory insults. While often neglected clinically, because there are no randomized studies to prove it, clinicians should each day seek doggedly to minimize or eliminate every factor that might promote ongoing inflammation.

Ultimately, the author believes---without definitive evidence---that if clinicians attend to these two items carefully and with perseverance, time is on our side and most patients recover, usually without developing fibroproliferative ARDS. As the inflammatory processes that initiated lung damage and attenuate hypoxic pulmonary vasoconstriction (HPV) are treated, shunt will decrease with time. Some patients with very severe ARDS demonstrate steal of blood whereby inflammation leads to nitric oxide-mediated vasodilation of the most diseased segments, and pathophysiologic shunt can significantly exceed the anatomic shunt fraction. But with less inflammation and return of HPV, blood is slowly redistributed to well-ventilated segments and oxygenation improves (provided initiating insults are addressed and additional insults are not introduced).[11,12]

## Mechanical Ventilation

While cardiogenic pulmonary edema often responds rapidly to preload reduction (e.g. diuretics, nitrates, non-invasive positive pressure ventilation; NIV) and interventions to improve left heart function, noncardiogenic pulmonary edema that typifies ARDS does not. The early, exudative phase is marked by a high shunt fraction typified by poor response to high concentrations of inspired oxygen (e.g. 100% non-rebreather face mask). While two small studies suggest that carefully selected immuno-incompetent patients with hypoxemic respiratory failure (which includes both ARDS and other illnesses) may respond to NIV, it should be used with caution and for short durations even in such patients if rapid clinical improvement (i.e. over minutes or hours) is not noted.[13,14] It should be used with extreme caution in other ARDS patient groups as its use has been associated with excessive mortality.[15]

The mainstay of supportive management of ARDS is invasive positive pressure ventilation (PPV). Obviously positive pressure does not treat flooding, but it does recruit atelectatic lung, thereby reducing the shunt fraction. Since exhalation is the lowest pressure during tidal breathing on PPV, the risk of derecruitment is greatest during exhalation. Note also, however, that during cough and dyssynchrony with PPV, derecruitment is also common.

Decades of animal and human research can be distilled to the following: ventilators do not cure ARDS but they can kill patients with ARDS if not used properly.[2] PPV is a bridge to buy doctors time to reverse the inflammatory stimuli that initiated and promote ARDS and time for the lung to heal. Early landmark studies demonstrated that the lungs of patients with ARDS were “small lungs,” i.e. large areas of gravitationally dependent atelectasis, a border zone of sometimes-open, sometimes-closed alveoli, and a gravitationally independent region of a well-ventilated lung.[8] PPV---specifically PEEP---can be used to maintain open some of the border-zone areas. However, if one applies “normal” tidal volumes to the remaining good alveoli they are over-distended and injured. This hypothesis was tested in the landmark trial in which patients with ALI/ARDS were randomized to receive volume-cycled ventilation that commenced at a tidal volume of 6 ml/kg but was soon titrated to achieve a distending (surrogate=“plateau”) pressure of ≤30 cmH_2_O or initially 12 ml/kg titrated to achieve a plateau pressure of ≤50 cmH_2_O. The mortality in the low tidal volume group was 31% compared to the more historically consistent 40% noted in the high tidal volume group.[2]

Many novices fixate on the weight-based prescription of this trial. In reality, mechanical ventilation should be customized to each patient’s unique pathophysiology which may change from hour-to-hour and often changes from day-to-day, while the weight does not reflect changes in lung pathophysiology. A properly-measured (i.e. static when the patient’s muscles are not active) plateau pressure is the best surrogate for over- (>30 cmH_2_O) and under-distension (<15 cmH_2_O) of the lung. Intubation followed by institution of 6--7 ml/kg is a reasonable starting point, but the clinician should remain at the bedside when ARDS is suspected to titrate ventilator settings:[2]

1. Titrate *V* _t_to a *P*_plt_ <30 cmH_2_O (i.e. 25--30 cmH_2_O) whenever possible.[2] For patients with extreme obesity, extreme PEEP requirements--->15 cmH_2_O---or other restrictive diatheses, higher plateaus are acceptable but only if absolutely necessary, and should rarely exceed 35 cmH_2_O (never ≥40 cmH_2_O).
2. If hypoxemia (O_2_sat<90%) persists despite 100% oxygen and PEEP = 5 cmH_2_O, increase PEEP in increments of 2-3 cmH_2_O and/or simply increase to 12--15 cmH_2_O all at once if ARDS is strongly suspected (this is the mean level of PEEP required by patients with ARDS). Titrate up PEEP 2-3 cmH_2_O every 3-5 min, as you titrate down FiO_2_to 60% (keeping O_2_sat≥90%). Note that as PEEP is increased, tidal volume must be reduced to maintain the *P*_plt_ <30 cmH_2_O. PEEP will increase lung recruitment, but it may not happen all at once and the goal is NOT to barotraumatize “healthy” lung segments with excessive distending pressures (reflected by *P*_plt_). The “best PEEP” remains unresolved.[16] It should be pointed out that PEEP has a number of effects. The salutatory effect of PEEP on oxygen results from its effect to maintain open formerly atelectatic lung units, i.e. to reduce the anatomic shunt fraction.[17] However, PEEP has at least two negative effects. First, in preload-deficient patients, it reduces cardiac output by reducing venous return. Second, PEEP increases mean alveolar pressure, and if in excess of capillary pressure, it may promote dead space and shunting of blood to poorly ventilated regions where alveolar hypertension is not present.[17] Which effect of PEEP predominates in any given patient varies from minute-to-minute and day-to-day. But, if upwards of PEEP = 20--25 cmH_2_O does not allow reductions to “non-toxic” fraction inspired oxygen (FiO_2_) of ≤60%, a recruitment maneuver; 2--3 min of methods used to recruit atelectatic alveoli, may be helpful transiently.[18] Recruitment maneuvers have never been proven to improve outcomes of critically ill patients, but rather are “tools” in the arsenal of salvage therapies (see below). Another option is simply to wait – there has never been definitive data that oxygen toxicity occurs in humans, and often recruitment improves with time (and application of PEEP) allowing decrements of FiO_2_. Moreover, if sepsis and other inflammatory processes are controlled, HPV is likely to improve oxygenation with time (assuming that precipitants of ARDS are treated/controlled---see below).

Patients with very severe ARDS may require high PEEP (as high as 25 cmH_2_O) and very low tidal volumes (as low as 200 ml) to achieve this healing-promoting strategy. In such patients, synchrony with the ventilator is essential since PEEP>10 cmH_2_O is difficult to provide if the patient is actively breathing to “combat the PEEP.” Deep sedation and muscle relaxation may be required to promote safe synchrony and allow PEEP to work in the most severe cases of ARDS.[19] Muscle relaxation should be used in the lowest doses and shortest durations (e.g. only “PRN” with dyssynchrony) possibly to reduce the likelihood of catastrophic neuromyopathies/quadriparesis that can result with prolonged relaxation.[20] But in rare cases, muscle relaxation is required to buy time for healing, even at the expense of this risk. Similarly, while daily awakening promotes recovery in other groups, it must be performed very carefully in patients with severe ARDS (as excessive awakening and subsequent dyssynchrony can promote prolonged derecruitment and hypoxemia).[21] Not infrequently, tidal volumes are so low, even at respiratory rates of 30/min (beware of going much higher), alveolar ventilation is insufficient, CO_2_ rises and pH decreases. It has been demonstrated that such “permissive hypercapnia” is reasonably safe and bicarbonate infusions are seldom needed even for pH as low as 7.1.[2,22]

## Recovery Phase

While specific PEEP strategies have failed to provide convincing results, ventilation can be conceived as occurring in a more narrow envelope (of higher PEEP to reduce atelectasis and lower tidal volume to reduce VILI).[16] As the lung heals and FiO_2_ required to maintain oxygen saturation above 90% reaches 50%, PEEP can be reduced 2 cmH_2_O every 2-4 h as tolerated. But the “envelope” of ventilation (i.e. between PEEP and plateau pressure) must move back toward normal, lest insufficient delivered tidal volume promotes atelectasis. So as PEEP can come down---as the lung heals and more alveoli become available at lower PEEP---tidal volume will need to be titrated up (again, using plateau 25-30 cmH_2_O as the set-point). Thus conceived, the ventilatory strategy during recovery can be viewed as the mirror image of escalation described above.

Finally, some argue that pressure-cycled ventilation is reasonable to try when volume-cycled ventilation fails. First, in my experience (17+ years), the ARDSnet approach has rarely (if ever) failed to achieve sufficient oxygenation. Second, pressure-controlled ventilation does not cycle on the plateau pressure but rather on the peak pressure (which changes dramatically even with secretions in the endotracheal tube). Third, there are no data to suggest that pressure-controlled ventilation improves outcomes. In fact, in contrast, pressure-controlled ventilation may be associated with higher morbidity.[23] Inverse ratio ventilation has also been offered as a means of improving lung recruitment, but at the cost of higher mean airway pressures---with no evidence of survival benefit. As of November 2010, the ARDSnet strategy is the only PPV approach shown to improve patient outcomes.[2] Until prospective randomized studies demonstrate benefits, pressure-controlled ventilation, inverse ratio ventilation, pressure-release ventilation, and high-frequency ventilation should be applied carefully, preferably in carefully conducted clinical trials.

## Fluid Management

Animal studies suggest that fluid fluxes are less when ARDS subjects are kept “as dry as possible.”[24] While another study of the ARDSnet study group failed to demonstrate a mortality benefit with fluid restriction, the number of ventilator-free days was reduced by 2.5 days (i.e. 17%).[25] But again, there is great risk if this idea is applied inappropriately. Septic shock is the most common cause of ARDS and fluid restriction in cases of septic shock complicated by ARDS could increase mortality.[26] Accordingly, clinicians must be particularly careful in such patients, filling (but not overfilling) during initial resuscitation and limiting fluid once hemodynamic stability is achieved.[27,28] I generally add a pressor earlier in resuscitation of septic patients whose course is complicated by severe pneumonia or ARDS.

Since many critically ill patients require large volumes to maintain organ perfusion in early phases of critical illness, great attention may be required to “retrieve” fluid as it rushes back to the intravascular space (and often into the lung) during recovery. Accordingly, as soon as a patient is stable and no longer requires pressors to maintain their circulation, clinicians should seek to retrieve fluids via diuresis. The author routinely computes total cumulative inputs and, once circulation is stable off pressors and fluid infusions, diureses as fast as the intravascular space---measured by a stable or falling BUN--- will tolerate.[28] This approach awaits clinical testing, but is consistent with previously published studies.[24–27]

## Rescue Therapies

Although it appears homogenous on chest radiographs, ARDS heterogeneously affects regions of the lung: areas in dependent lung regions are atelectatic and/or flooded, while those in independent lung regions are often well recruited.[8] Accordingly, prone ventilation can be used to take advantage of this gravitational gradient, in cases that are refractory (PaO_2_:FiO_2_<100 on high PEEP) to the ARDSnet strategy. It has not been shown to improve outcomes, but if performed very carefully (securing tubes, lines and stabilizing the neck), it often improves oxygenation of patients who have refractory hypoxemia requiring high PEEP and FiO_2_>60% in the recumbent position.[29] Importantly, no study has addressed whether prone position improves outcomes in severe cases refractory to the ARDSnet approach (e.g. PaO_2_<60 mmHg on FiO_2_>60% and PEEP>15 cmH_2_O). Studies “flipped” patients prone-recumbent based on arbitrary times, rather than on their physiology. In reality, proning is not required routinely for patients with mild or even moderate ARDS, so published studies do not settle whether proning has a place in management of severe ARDS---as a form of “rescue therapy.”

The same can be said for the utility of nitric oxide (NO) in ARDS. While administration of nitric-oxide up to 28 ppm, which promotes greater blood flow to well-ventilated segments, may reduce shunt fraction and improve oxygenation, its effects on outcomes and role in management of adult ARDS is not clear.[30] Again, this therapy should not be needed for the vast majority of patients with ARDS; a very large “*n*” would be required to demonstrate a difference of NO-therapy if one existed among mild-moderate cases. Accordingly, the author contends that the role of nitric oxide for severe cases (see above) remains an unanswered question.

H1N1 influenza caused a particularly severe form of ARDS that was reported to be refractory to volume-controlled ventilation using the principles reviewed herein. Extracorporeal membrane oxygenation (ECMO) was used in selected patients with promising results, but its efficacy awaits testing in a prospective randomized study.[31]

It is also worth restating that ARDS may be thought of as a multifactorial disease whose etiology may involve several mechanisms initially and whose pathogenesis may change with time. For example, a patient with ARDS related to overwhelming septic shock might have infection as the initial precipitant, but with treatment of the infection, other factors (e.g. VILI, nosocomial infections, medications, TRALI, etc) may become the pathophysiologic stimuli for it to continue rather than remit. Clinicians should thus use the ARDSnet strategy and seek to minimize the likelihood (i.e. identify and treat) other pro-inflammatory events propagate the injury. Along these lines, patients whose gas exchange do not improve by days 7--8 despite ruling out of the processes listed above are candidates for corticosteroids (methylprednisilone 2 mg/kg/d; author suggests stopping after 7 d if no salutatory effects).[32] However, in the largest study to date of such patients randomized to steroids or routine care, there was no difference in outcomes.[33] Accordingly, steroids are not proven to change the course of fibroproliferative ARDS, but may have a role in cases that are not resolving in 7--10 days and no other pro-inflammatory precipitant can be identified. Otherwise, a multitude of various anti-inflammatory therapies, from fish oils to activated protein C have failed to change the course of this enigmatic disease.

## Conclusions

While bench and clinical investigations have yielded a wealth of information, to date there is only one management approach demonstrated to attenuate mortality of patients with ARDS. Clinicians should understand the pathogenesis of this disease, and simultaneously administer an evidence- and common sense-based approach until future breakthroughs add to our armamentarium.[2]
